# Tris(1,10-phenanthroline)cobalt(II) bis­(trichloro­acetate)

**DOI:** 10.1107/S160053681102410X

**Published:** 2011-06-25

**Authors:** Li-Min Li, Yu-Feng Li, Li Liu, Zeng-Hui Zhang

**Affiliations:** a Microscale Science Institute, Department of Chemistry and Chemical Engineering, Weifang University, Weifang 261061, People’s Republic of China; b Department of Chemistry and Chemical Engineering, Weifang University, Weifang 261061, People’s Republic of China

## Abstract

In the title complex, [Co(C_12_H_8_N_2_)_3_](C_2_Cl_3_O_2_)_2_, the Co^II^ ion lies on a twofold rotation axis and is coordinated by six N atoms from three bis-chelating 1,10-phenanthroline ligands in a distorted octa­hedral environment. The crystal structure is stabilized by weak inter­molecular C—H⋯O hydrogen bonds.

## Related literature

For background to metal-organic framework coordination polymers, see: Chen *et al.* (2001 ▶); Fang *et al.* (2005 ▶). For a related structure, see: Harding *et al.* (2008 ▶).
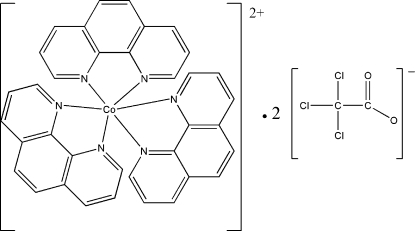


## Experimental

### 

#### Crystal data

[Co(C_12_H_8_N_2_)_3_](C_2_Cl_3_O_2_)_2_
*M* *_r_* = 924.28
Monoclinic, 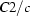

*a* = 18.367 (4) Å
*b* = 10.753 (2) Å
*c* = 19.020 (4) Å
β = 100.94 (3)°
*V* = 3688.2 (13) Å^3^
*Z* = 4
Mo *K*α radiation
μ = 0.95 mm^−1^
*T* = 293 K
0.26 × 0.20 × 0.12 mm

#### Data collection

Bruker SMART CCD diffractometer
Absorption correction: multi-scan (*SADABS*; Sheldrick, 1996 ▶) *T*_min_ = 0.837, *T*_max_ = 0.923
17083 measured reflections
4215 independent reflections
3364 reflections with *I* > 2σ(*I*)
*R*_int_ = 0.092

#### Refinement

*R*[*F* ^2^ > 2σ(*F* ^2^)] = 0.048
*wR*(*F* ^2^) = 0.142
*S* = 0.89
4215 reflections
258 parameters
H-atom parameters constrained
Δρ_max_ = 0.81 e Å^−3^
Δρ_min_ = −0.45 e Å^−3^

### 

Data collection: *SMART* (Bruker, 1997 ▶); cell refinement: *SAINT* (Bruker, 1997 ▶); data reduction: *SAINT*; program(s) used to solve structure: *SHELXS97* (Sheldrick, 2008 ▶); program(s) used to refine structure: *SHELXL97* (Sheldrick, 2008 ▶); molecular graphics: *SHELXTL* (Sheldrick, 2008 ▶); software used to prepare material for publication: *SHELXTL*.

## Supplementary Material

Crystal structure: contains datablock(s) global, I. DOI: 10.1107/S160053681102410X/lh5253sup1.cif

Structure factors: contains datablock(s) I. DOI: 10.1107/S160053681102410X/lh5253Isup2.hkl

Additional supplementary materials:  crystallographic information; 3D view; checkCIF report

## Figures and Tables

**Table 1:** Hydrogen-bond geometry (Å, °)

*D*—H⋯*A*	*D*—H	H⋯*A*	*D*⋯*A*	*D*—H⋯*A*
C9—H9*A*⋯O1^i^	0.93	2.54	3.401 (3)	154
C10—H10*A*⋯O2^ii^	0.93	2.35	3.120 (3)	140
C13—H13*A*⋯O1^iii^	0.93	2.28	3.004 (3)	134
C14—H14*A*⋯O1^iv^	0.93	2.60	3.455 (3)	154
C15—H15*A*⋯O2^iv^	0.93	2.56	3.266 (3)	133

Symmetry codes: (i) 
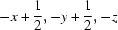
; (ii) 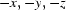; (iii) 
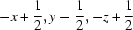
; (iv) 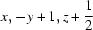.

**Fig. 1.**
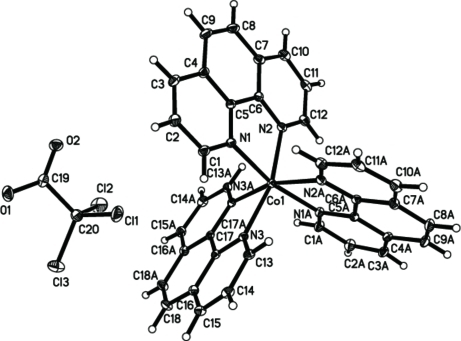
The molecular structure of the title compound showing 30% probability displacement ellipsoids (symmetry code: (A) -x, y, -z + 1/2). Only the unique anion is shown.

## supplementary crystallographic information

### Comment

Metal-organic framework coordination polymers have attracted tremendous attention because of their molecular topologies and their potentially useful ionexchange, adsorption, catalytic and magnetic properties (Chen *et al.*, 2001; Fang *et al.*,2005). As part of our search for new complexes of this type, we synthesized the title compound and report its crystal structure herein.

The molecular structure of the title complex is shown in Fig. 1. The Co^II^ ion lies on a twofold rotation axis and is coordinated by six N atoms of three bis-chelating 1,10-phenanthroline ligands in a distorted octahedral environment. The Co—N bond lengths are in agreement with those reported for a related complex (Harding *et al.*, 2008). The crystal structure is stabilized by weak intermolecular C—H···O hydrogen bonds.

### Experimental

The title compound was obtained by adding 1,10-phenanthroline (3 mmol) dropwise to a solution of cobalt(II) trichloroacetic acid (1 mmol) in ethanol (20 ml). The solution was stirred for 1 h at room temperature. After a few days block-shaped crystals were formed from the yellow solution.

### Refinement

H atoms were fixed geometrically and allowed to ride on their attached atoms, with C—H = 0.93Å and *U*_iso_(H) = 1.2*U*_eq_(C).

### Crystal data

**Table:**

[Co(C_12_H_8_N_2_)_3_](C_2_Cl_3_O_2_)_2_	*F*(000) = 1868
*M**_r_* = 924.28	*D*_x_ = 1.665 Mg m^−^^3^
Monoclinic, *C*2/*c*	Mo *K*α radiation, λ = 0.71073 Å
Hall symbol: -C 2yc	Cell parameters from 3364 reflections
*a* = 18.367 (4) Å	θ = 3.3–27.5°
*b* = 10.753 (2) Å	µ = 0.95 mm^−^^1^
*c* = 19.020 (4) Å	*T* = 293 K
β = 100.94 (3)°	Block, yellow
*V* = 3688.2 (13) Å^3^	0.26 × 0.20 × 0.12 mm
*Z* = 4	

### Data collection

**Table:**

Bruker SMART CCD diffractometer	4215 independent reflections
Radiation source: fine-focus sealed tube	3364 reflections with *I* > 2σ(*I*)
graphite	*R*_int_ = 0.092
Detector resolution: 9 pixels mm^-1^	θ_max_ = 27.5°, θ_min_ = 3.3°
φ and ω scans	*h* = −23→23
Absorption correction: multi-scan (*SADABS*; Sheldrick, 1996)	*k* = −13→13
*T*_min_ = 0.837, *T*_max_ = 0.923	*l* = −22→24
17083 measured reflections	

### Refinement

**Table:**

Refinement on *F*^2^	Primary atom site location: structure-invariant direct methods
Least-squares matrix: full	Secondary atom site location: difference Fourier map
*R*[*F*^2^ > 2σ(*F*^2^)] = 0.048	Hydrogen site location: inferred from neighbouring sites
*wR*(*F*^2^) = 0.142	H-atom parameters constrained
*S* = 0.89	*w* = 1/[σ^2^(*F*_o_^2^) + (0.1*P*)^2^] where *P* = (*F*_o_^2^ + 2*F*_c_^2^)/3
4215 reflections	(Δ/σ)_max_ < 0.001
258 parameters	Δρ_max_ = 0.81 e Å^−^^3^
0 restraints	Δρ_min_ = −0.45 e Å^−^^3^

### Special details

**Table:**

Geometry. All e.s.d.'s (except the e.s.d. in the dihedral angle between two l.s. planes) are estimated using the full covariance matrix. The cell e.s.d.'s are taken into account individually in the estimation of e.s.d.'s in distances, angles and torsion angles; correlations between e.s.d.'s in cell parameters are only used when they are defined by crystal symmetry. An approximate (isotropic) treatment of cell e.s.d.'s is used for estimating e.s.d.'s involving l.s. planes.
Refinement. Refinement of *F*^2^ against ALL reflections. The weighted *R*-factor *wR* and goodness of fit *S* are based on *F*^2^, conventional *R*-factors *R* are based on *F*, with *F* set to zero for negative *F*^2^. The threshold expression of *F*^2^ > σ(*F*^2^) is used only for calculating *R*-factors(gt) *etc*. and is not relevant to the choice of reflections for refinement. *R*-factors based on *F*^2^ are statistically about twice as large as those based on *F*, and *R*- factors based on ALL data will be even larger.

### Fractional atomic coordinates and isotropic or equivalent isotropic displacement parameters (Å^2^)

**Table:**

	*x*	*y*	*z*	*U*_iso_*/*U*_eq_	
Co1	0.0000	0.08051 (4)	0.2500	0.01371 (15)	
N1	0.08711 (11)	0.05924 (18)	0.19106 (10)	0.0166 (4)	
N2	−0.04713 (11)	−0.05011 (17)	0.16840 (10)	0.0171 (4)	
N3	0.05538 (10)	0.23619 (17)	0.30672 (9)	0.0139 (4)	
C1	0.15367 (13)	0.1115 (2)	0.20262 (13)	0.0219 (5)	
H1A	0.1671	0.1614	0.2429	0.026*	
C2	0.20463 (14)	0.0959 (2)	0.15766 (14)	0.0244 (5)	
H2A	0.2511	0.1330	0.1686	0.029*	
C3	0.18537 (14)	0.0252 (2)	0.09727 (13)	0.0230 (5)	
H3A	0.2174	0.0175	0.0651	0.028*	
C4	0.11654 (13)	−0.0354 (2)	0.08449 (12)	0.0199 (5)	
C5	0.06900 (12)	−0.0155 (2)	0.13273 (11)	0.0163 (4)	
C6	−0.00237 (13)	−0.0750 (2)	0.12096 (12)	0.0158 (4)	
C7	−0.02363 (13)	−0.1525 (2)	0.06160 (12)	0.0209 (5)	
C8	0.02684 (15)	−0.1718 (2)	0.01384 (13)	0.0264 (5)	
H8A	0.0134	−0.2243	−0.0253	0.032*	
C9	0.09337 (15)	−0.1153 (3)	0.02451 (13)	0.0260 (5)	
H9A	0.1248	−0.1286	−0.0077	0.031*	
C10	−0.09434 (13)	−0.2060 (2)	0.05130 (13)	0.0234 (5)	
H10A	−0.1101	−0.2593	0.0129	0.028*	
C11	−0.13996 (13)	−0.1790 (2)	0.09837 (14)	0.0239 (5)	
H11A	−0.1876	−0.2120	0.0918	0.029*	
C12	−0.11401 (13)	−0.1013 (2)	0.15618 (13)	0.0209 (5)	
H12A	−0.1453	−0.0843	0.1881	0.025*	
C13	0.11155 (12)	0.2351 (2)	0.36144 (12)	0.0179 (5)	
H13A	0.1308	0.1586	0.3786	0.022*	
C14	0.14369 (13)	0.3426 (2)	0.39495 (12)	0.0208 (5)	
H14A	0.1832	0.3371	0.4335	0.025*	
C15	0.11645 (13)	0.4560 (2)	0.37044 (12)	0.0217 (5)	
H15A	0.1360	0.5285	0.3931	0.026*	
C16	0.05858 (12)	0.4615 (2)	0.31049 (12)	0.0173 (5)	
C17	0.02949 (12)	0.3489 (2)	0.28041 (11)	0.0149 (4)	
C18	0.02748 (14)	0.5754 (2)	0.27896 (14)	0.0225 (5)	
H18A	0.0457	0.6508	0.2989	0.027*	
Cl1	0.18800 (5)	0.41813 (7)	0.18548 (4)	0.0414 (2)	
Cl2	0.06670 (4)	0.51120 (9)	0.07856 (5)	0.0454 (2)	
Cl3	0.17181 (4)	0.67933 (6)	0.15928 (4)	0.0368 (2)	
O1	0.25779 (11)	0.59450 (19)	0.05738 (11)	0.0341 (5)	
O2	0.19590 (11)	0.41976 (17)	0.02240 (10)	0.0292 (4)	
C20	0.16126 (14)	0.5325 (2)	0.11741 (13)	0.0245 (5)	
C19	0.21116 (12)	0.5134 (2)	0.05881 (12)	0.0215 (5)	

### Atomic displacement parameters (Å^2^)

**Table:**

	*U*^11^	*U*^22^	*U*^33^	*U*^12^	*U*^13^	*U*^23^
Co1	0.0146 (2)	0.0140 (2)	0.0126 (2)	0.000	0.00274 (17)	0.000
N1	0.0169 (9)	0.0178 (9)	0.0155 (9)	−0.0009 (7)	0.0041 (8)	−0.0021 (7)
N2	0.0195 (9)	0.0134 (9)	0.0174 (9)	0.0003 (7)	0.0010 (8)	−0.0002 (7)
N3	0.0144 (8)	0.0157 (9)	0.0111 (8)	−0.0005 (7)	0.0013 (7)	−0.0017 (6)
C1	0.0217 (12)	0.0256 (12)	0.0189 (12)	−0.0045 (10)	0.0054 (10)	−0.0047 (9)
C2	0.0184 (11)	0.0283 (13)	0.0276 (13)	−0.0051 (10)	0.0071 (10)	−0.0035 (10)
C3	0.0237 (12)	0.0279 (13)	0.0198 (12)	0.0033 (10)	0.0101 (10)	0.0014 (9)
C4	0.0246 (12)	0.0207 (11)	0.0153 (11)	0.0041 (9)	0.0060 (9)	0.0025 (8)
C5	0.0186 (10)	0.0160 (10)	0.0140 (10)	0.0028 (9)	0.0025 (9)	0.0009 (8)
C6	0.0180 (10)	0.0154 (10)	0.0132 (10)	0.0015 (8)	0.0009 (9)	0.0018 (8)
C7	0.0260 (12)	0.0181 (11)	0.0164 (11)	0.0019 (9)	−0.0018 (10)	−0.0018 (8)
C8	0.0363 (14)	0.0254 (13)	0.0174 (12)	0.0043 (11)	0.0047 (11)	−0.0051 (9)
C9	0.0323 (13)	0.0300 (13)	0.0179 (12)	0.0043 (11)	0.0104 (11)	−0.0027 (10)
C10	0.0255 (11)	0.0203 (11)	0.0204 (12)	−0.0014 (10)	−0.0059 (10)	−0.0024 (9)
C11	0.0206 (11)	0.0164 (12)	0.0318 (14)	−0.0016 (9)	−0.0022 (10)	0.0017 (9)
C12	0.0176 (11)	0.0179 (11)	0.0264 (13)	−0.0005 (9)	0.0023 (10)	0.0006 (9)
C13	0.0184 (10)	0.0208 (11)	0.0138 (11)	0.0038 (9)	0.0009 (9)	−0.0003 (8)
C14	0.0160 (10)	0.0305 (13)	0.0135 (10)	−0.0014 (9)	−0.0033 (9)	−0.0025 (9)
C15	0.0248 (12)	0.0210 (12)	0.0191 (11)	−0.0033 (10)	0.0041 (10)	−0.0047 (9)
C16	0.0149 (10)	0.0187 (11)	0.0184 (11)	−0.0006 (9)	0.0037 (9)	−0.0009 (8)
C17	0.0147 (10)	0.0169 (11)	0.0140 (10)	0.0009 (8)	0.0053 (9)	−0.0002 (8)
C18	0.0270 (12)	0.0141 (11)	0.0247 (12)	−0.0015 (9)	0.0007 (10)	−0.0019 (9)
Cl1	0.0599 (5)	0.0325 (4)	0.0382 (4)	0.0076 (3)	0.0255 (4)	0.0144 (3)
Cl2	0.0190 (3)	0.0604 (5)	0.0581 (5)	−0.0049 (3)	0.0110 (3)	−0.0155 (4)
Cl3	0.0469 (4)	0.0273 (4)	0.0337 (4)	0.0072 (3)	0.0007 (3)	−0.0071 (3)
O1	0.0268 (10)	0.0393 (12)	0.0367 (11)	−0.0156 (8)	0.0074 (9)	−0.0009 (8)
O2	0.0271 (10)	0.0328 (10)	0.0277 (10)	−0.0036 (8)	0.0049 (8)	−0.0083 (7)
C20	0.0221 (11)	0.0250 (13)	0.0258 (13)	−0.0022 (10)	0.0033 (10)	−0.0008 (9)
C19	0.0148 (10)	0.0309 (13)	0.0176 (11)	−0.0020 (9)	0.0001 (9)	0.0034 (9)

### Geometric parameters (Å, °)

**Table:**

Co1—N1^i^	2.1330 (19)	C8—H8A	0.9300
Co1—N1	2.1330 (19)	C9—H9A	0.9300
Co1—N3	2.1411 (18)	C10—C11	1.368 (4)
Co1—N3^i^	2.1411 (18)	C10—H10A	0.9300
Co1—N2	2.1497 (19)	C11—C12	1.391 (3)
Co1—N2^i^	2.1497 (19)	C11—H11A	0.9300
N1—C1	1.325 (3)	C12—H12A	0.9300
N1—C5	1.359 (3)	C13—C14	1.396 (3)
N2—C12	1.326 (3)	C13—H13A	0.9300
N2—C6	1.358 (3)	C14—C15	1.366 (3)
N3—C13	1.319 (3)	C14—H14A	0.9300
N3—C17	1.362 (3)	C15—C16	1.405 (3)
C1—C2	1.393 (3)	C15—H15A	0.9300
C1—H1A	0.9300	C16—C17	1.401 (3)
C2—C3	1.366 (4)	C16—C18	1.434 (3)
C2—H2A	0.9300	C17—C17^i^	1.427 (4)
C3—C4	1.402 (3)	C18—C18^i^	1.345 (5)
C3—H3A	0.9300	C18—H18A	0.9300
C4—C5	1.398 (3)	Cl1—C20	1.786 (3)
C4—C9	1.428 (3)	Cl2—C20	1.769 (3)
C5—C6	1.438 (3)	Cl3—C20	1.762 (3)
C6—C7	1.398 (3)	O1—C19	1.226 (3)
C7—C10	1.400 (3)	O2—C19	1.224 (3)
C7—C8	1.431 (3)	C20—C19	1.585 (3)
C8—C9	1.345 (4)		
			
N1^i^—Co1—N1	167.69 (10)	C10—C7—C8	123.2 (2)
N1^i^—Co1—N3	98.69 (7)	C9—C8—C7	121.2 (2)
N1—Co1—N3	90.95 (7)	C9—C8—H8A	119.4
N1^i^—Co1—N3^i^	90.95 (7)	C7—C8—H8A	119.4
N1—Co1—N3^i^	98.69 (7)	C8—C9—C4	121.1 (2)
N3—Co1—N3^i^	77.14 (10)	C8—C9—H9A	119.4
N1^i^—Co1—N2	94.01 (8)	C4—C9—H9A	119.4
N1—Co1—N2	77.87 (7)	C11—C10—C7	119.2 (2)
N3—Co1—N2	164.22 (7)	C11—C10—H10A	120.4
N3^i^—Co1—N2	93.40 (7)	C7—C10—H10A	120.4
N1^i^—Co1—N2^i^	77.87 (7)	C10—C11—C12	119.0 (2)
N1—Co1—N2^i^	94.01 (8)	C10—C11—H11A	120.5
N3—Co1—N2^i^	93.40 (7)	C12—C11—H11A	120.5
N3^i^—Co1—N2^i^	164.22 (7)	N2—C12—C11	123.7 (2)
N2—Co1—N2^i^	98.40 (10)	N2—C12—H12A	118.1
C1—N1—C5	117.5 (2)	C11—C12—H12A	118.1
C1—N1—Co1	128.87 (16)	N3—C13—C14	123.5 (2)
C5—N1—Co1	113.61 (15)	N3—C13—H13A	118.2
C12—N2—C6	117.3 (2)	C14—C13—H13A	118.2
C12—N2—Co1	129.10 (17)	C15—C14—C13	119.2 (2)
C6—N2—Co1	113.39 (15)	C15—C14—H14A	120.4
C13—N3—C17	117.65 (19)	C13—C14—H14A	120.4
C13—N3—Co1	128.03 (16)	C14—C15—C16	119.1 (2)
C17—N3—Co1	114.28 (14)	C14—C15—H15A	120.4
N1—C1—C2	123.6 (2)	C16—C15—H15A	120.4
N1—C1—H1A	118.2	C17—C16—C15	117.8 (2)
C2—C1—H1A	118.2	C17—C16—C18	118.5 (2)
C3—C2—C1	119.1 (2)	C15—C16—C18	123.8 (2)
C3—C2—H2A	120.4	N3—C17—C16	122.7 (2)
C1—C2—H2A	120.4	N3—C17—C17^i^	117.15 (12)
C2—C3—C4	119.0 (2)	C16—C17—C17^i^	120.20 (13)
C2—C3—H3A	120.5	C18^i^—C18—C16	121.32 (14)
C4—C3—H3A	120.5	C18^i^—C18—H18A	119.3
C5—C4—C3	118.0 (2)	C16—C18—H18A	119.3
C5—C4—C9	119.2 (2)	C19—C20—Cl3	113.91 (17)
C3—C4—C9	122.7 (2)	C19—C20—Cl2	110.03 (16)
N1—C5—C4	122.6 (2)	Cl3—C20—Cl2	108.66 (14)
N1—C5—C6	117.7 (2)	C19—C20—Cl1	107.71 (17)
C4—C5—C6	119.6 (2)	Cl3—C20—Cl1	107.33 (13)
N2—C6—C7	122.9 (2)	Cl2—C20—Cl1	109.08 (14)
N2—C6—C5	117.3 (2)	O2—C19—O1	131.2 (2)
C7—C6—C5	119.9 (2)	O2—C19—C20	113.8 (2)
C6—C7—C10	117.9 (2)	O1—C19—C20	115.0 (2)
C6—C7—C8	118.9 (2)		

Symmetry codes: (i) −*x*, *y*, −*z*+1/2.

### Hydrogen-bond geometry (Å, °)

**Table:**

*D*—H···*A*	*D*—H	H···*A*	*D*···*A*	*D*—H···*A*
C9—H9A···O1^ii^	0.93	2.54	3.401 (3)	154
C10—H10A···O2^iii^	0.93	2.35	3.120 (3)	140
C13—H13A···O1^iv^	0.93	2.28	3.004 (3)	134
C14—H14A···O1^v^	0.93	2.60	3.455 (3)	154
C15—H15A···O2^v^	0.93	2.56	3.266 (3)	133

Symmetry codes: (ii) −*x*+1/2, −*y*+1/2, −*z*; (iii) −*x*, −*y*, −*z*; (iv) −*x*+1/2, *y*−1/2, −*z*+1/2; (v) *x*, −*y*+1, *z*+1/2.
